# Utilizing primary care to engage underserved patients in a psychological intervention for chronic pain

**DOI:** 10.1017/S1463423624000471

**Published:** 2024-10-25

**Authors:** Lisa R. Miller-Matero, Leah M. Hecht, Lyubov Gavrilova, Brittany Haage, Kirsti Autio, Erin T. Tobin, Brian K. Ahmedani

**Affiliations:** 1 Henry Ford Health, Behavioral Health, Detroit, MI, USA; 2 Henry Ford Health, Center for Health Policy & Health Services Research, Detroit, MI, USA; 3 Michigan State University, East Lansing, MI, USA; 4 Henry Ford Health, Public Health Sciences, Detroit, MI, USA; 5 Henry Ford Health, Internal Medicine, Detroit, MI, USA

**Keywords:** chronic pain, integrated care, primary care, psychological intervention, underserved

## Abstract

**Background:**

Although psychological interventions can be used to improve chronic pain management, underserved individuals (i.e., racially minoritized and socioeconomically disadvantaged) may be less likely to engage in such services. The purpose of this study was to examine whether offering a psychological intervention for chronic pain in a primary care clinic could be a method in which to successfully engage underserved patients.

**Methods:**

There were 220 patients with chronic pain in a primary care clinic located in a socioeconomically and racially diverse city who were approached to discuss enrolment in a pilot randomized controlled trial of a five-session psychological intervention for chronic pain. Patients were introduced to the study by their primary care provider using the warm handoff model. We compared whether there were sociodemographic differences between those who enrolled in the study and those who declined to enrol.

**Results:**

There were no differences between those who enrolled and those who declined enrolment with regard to race, age, insurance type, and household income. However, females were more likely to enrol in the study compared to males.

**Conclusions:**

Recruiting patients to participate in a trial of a psychological intervention for chronic pain in a primary care clinic appeared to be effective for engaging Black patients, patients with lower income, and those with government insurance. Thus, offering a psychological intervention for chronic pain in a primary care clinic may encourage engagement among racially minoritized individuals and those with lower socioeconomic status.

## Introduction

Chronic pain is a common medical condition, estimated to affect nearly one-third of Americans (Johannes *et al.*, 2010). The biopsychosocial model of pain suggests that psychological factors impact the experience of pain (Gatchel *et al.*, 2007). As such, psychological interventions for pain have been developed, including cognitive-behavioural therapy, acceptance and commitment therapy, and mindfulness meditation. The treatment model for cognitive-behavioural therapy for pain focuses on adjusting patients’ physical experience of pain, catastrophic thoughts, and maladaptive behaviours (Jensen *et al.*, 1994; Wetherell *et al.*, 2011). Mindfulness mediation teaches patients to observe their pain as only physical sensations, without trying to use distractions to escape any unpleasant feelings that may arise (Kabat-Zinn, 1982). Acceptance and commitment therapy extends upon some of the concepts in a mindfulness approach and teaches a patient how to experience painful physical sensations without trying to change them (Wetherell *et al.*, 2011). Patients learn that nonjudgmental acceptance of their condition can bring more relief than attempts to fight off sensations, which can lead to greater distress. These psychological interventions have led to improvements in pain severity, pain interference, and pain-related distress in patients with chronic pain (Keefe *et al.*, 1992; McCracken *et al.*, 2004; Zautra *et al.*, 2008; Vowles *et al.*, 2009), thus is a treatment option beyond traditional treatment methods such as medication and physical therapy.

Although patients with chronic pain experience depression and anxiety at 2–3 times the rate of the general population (McWilliams *et al.*, 2003; Miller & Cano, 2009), underserved populations (i.e., racial minorities and socioeconomically disadvantaged) appear to be at greater risk for experiencing pain-related distress. For example, there are cultural differences to the way certain groups experience pain, including adaptation and coping methods (Moore & Brodsgaard, 1999). Black patients with chronic pain are more likely to experience greater pain-related distress, including depression, and higher levels of pain unpleasantness, emotional response to pain, and greater disability, compared to White patients (Riley *et al.*, 2002; Fuentes *et al.*, 2007; Miller & Cano, 2009). Beliefs about pain could potentially explain why Black patients may experience higher levels of distress (Riley *et al.*, 2002). In addition to racial differences, patients with lower socioeconomic status (SES) are also at greater risk to experience pain-related distress. Those with lower SES tend to report lower quality of life, be diagnosed with mood disorders, and are more likely to develop a pain-related disability that interferes with daily functioning (Portenoy *et al.*, 2004; Fuentes *et al.*, 2007). Thus, Black patients and those with lower SES might especially benefit from a psychological approach for pain management.

Not only are underserved populations more likely to experience greater pain-related distress, they are also less likely to engage in psychological services, making these patients especially vulnerable to the effects of having a chronic pain condition. Specifically, racial and ethnic minorities are significantly less likely to engage in mental health services compared to White patients, even when bothered by their symptoms (Sussman *et al.*, 1987; Dobalian & Rivers, 2008). A low to middle SES background was also predictive of insufficient use of mental health services in African American and Hispanic populations (Dobalian & Rivers, 2008). There are likely multiple factors contributing to lower utilization of mental health services among underserved populations. First, underserved patients are less likely to access mental health services (Padgett *et al.*, 1994; US Surgeon General, 2001; Dobalian & Rivers, 2008). Lower access could be related to economic hardships that disproportionately affect minority groups (i.e., lack of adequate health insurance); however, even after controlling for SES, Black and Hispanic patients had a lower likelihood of engaging in outpatient mental health services than Whites (Padgett *et al.*, 1994). Second, cultural factors may influence willingness to engage in mental health services. Black patients may be less trusting of healthcare providers than White patients (Sussman *et al.*, 1987; Dobalian & Rivers, 2008) and may have higher levels of mental health stigma (Padgett *et al.*, 1994; Masuda *et al.*, 2012; Ward *et al.*, 2013), which could lead to lower engagement in mental health services (Givens *et al.*, 2007). Therefore, we need to identify methods to improve access to psychological interventions for underserved populations.

Integrating psychological interventions for chronic pain into primary care could improve utilization of these services and alleviate some of the disparities. Integrating psychological interventions for general mental health concerns in primary care appears to increase access to mental health services regardless of age, sex, or race (Miller-Matero *et al.*, 2015). This could be because integrating a mental health provider in primary care could remove logistical barriers (i.e., transportation, confusion with a new office) and lower stigma (Sadock *et al.*, 2014; Miller-Matero *et al.*, 2019). Although integrating mental health services in primary care appears to increase utilization for general mental health concerns, it is not clear whether this would improve access to psychological treatments for chronic pain in underserved populations. Importantly, preliminary work on implementing brief psychological interventions for chronic pain in primary care suggests improvements in patient outcomes (Beehler *et al.*, 2019; Miller-Matero *et al.*, 2021). Therefore, increasing utilization of these beneficial services could result in improved outcomes for patients who might not have otherwise sought out behavioural pain management services. The purpose of this study was to examine whether offering a psychological intervention for chronic pain in a primary care clinic could successfully engage underserved patients.

## Materials and methods

This study was a secondary analysis of a pilot randomized controlled trial for a psychological intervention for chronic pain delivered in primary care (Miller-Matero *et al.,* 2021). This intervention resulted in short- and longer-term benefits for pain and psychological functioning compared a treatment as usual control group (Miller-Matero *et al.,* 2021, 2022). To identify eligible patients for the study, psychology postdoctoral fellows reviewed electronic medical charts of patients who had an appointment with a primary care provider in a single, midwestern, urban primary care clinic in the United States. This clinic serves a diverse range of patients with regard to SES and race. Patients were eligible if they had a chronic, noncancer pain condition defined as lasting at least 3 months. Patients were excluded if they were currently engaging in behavioural health treatment (i.e., psychotherapy) or had cognitive impairment that would interfere with their ability to understand the content of the intervention (i.e., a diagnosis of a cognitive disorder). If patients met eligibility criteria upon chart review, the fellow would alert the primary care provider seeing the patient and the primary care provider would introduce the study to the patient during the visit. If the patient was interested in learning more, the fellow provided additional information to the patient. Patients were told that if they chose to enrol in the study, they would be randomized to either the control group (treatment as usual) or a five-session psychological intervention designed to improve management of chronic pain. The five sessions were composed of multiple evidence-based components including cognitive-behavioural, mindfulness, and acceptance-based strategies. Each session lasted approximately 45 minutes. Participants would complete an intake assessment, complete the five-session intervention (if randomized to the intervention group), and complete a post-assessment (approximately 5 weeks after baseline), and a 1-month and 6-month follow-up.

For the purposes of this study, participants were categorized as enrolled (i.e., eligible patients who completed informed consent and were randomized the intervention or control group) or declined to enrol (i.e., eligible patients who declined participation after the primary care provider told them about the study or declined to participate after the fellow explained the study further). There were 60 participants who enrolled in the study and 160 participants who declined to enrol. Thus, there were a total of 220 participants in this current study. This study was approved by the health system’s Institutional Review Board. Those that enrolled in the study provided informed consent to participate and the IRB waived consent to conduct chart reviews of those who did not enrol.

Retrospective chart reviews were conducted of all participants in the current study (*n* = 220). Age, sex, race, insurance type, and zip code were extracted from participants’ medical records. Zip code was used to estimate median household income from the Census Bureau. All analyses were conducted with SPSS version 25. Independents samples *t*-tests and chi-square analyses were conducted to determine whether there were differences in age, gender, race, insurance type, and household income between those who enrolled in the study and those who declined to enrol.

## Results

Participants who enrolled in the study were primarily female, Black, had government insurance, had a mean age of 62.17 years, and mean household income of $37,968.65 (Table 1). Those who declined to participate in the study were also primarily female, Black, had government insurance, had a mean age of 61.97 years, and mean household income of $35,160.28 (Table 1). There were no differences between those who enrolled and those who declined enrolment with regard to race, age, insurance type, and median household income. However, females were more likely to enrol in the study compared to males.

**Table 1.** Comparison of participants who enrolled and those who declined to enrol

	**Enrolled (** ***n*** **= 60)**		**Nonenrolled (** ***n*** **= 120)**			
	***M***	***SD***	***M***	***SD***	***t***	***p***
Age, years	62.17	12.68	61.97	13.51	−0.10	0.92
Household income	$37,968.65	$19,227.64	$35,160.28	$13,452.25	−1.04	0.30
	**%**	***n***	**%**	***n***	***X*** ^***2***^	***p***
**Sex**						
Female	62.5	100	78.3	47	4.93	0.03
Male	37.5	60	21.7	13		
**Race** ^**a**^						
Black	88.3	53	88.8	142	2.13	0.65
White	11.7	7	9.4	15		
Other			1.9	3		
**Insurance status** ^**b**^						
Government	65.0	39	71.2	114	0.25	0.61
Private	23.3	14	21.3	34		
Other	11.7	7	7.5	12		

a Chi-square analysis only compared Black and White participants.

b Chi-square analysis only compared those with government or private insurance.

## Discussion

This study sought to examine whether offering a psychological intervention for chronic pain in primary care may facilitate care to underserved individuals by examining rates of study enrolment by age, race, gender, insurance type, and income. Results from this study showed that enrolment status was not associated with age, race, insurance type, or income, suggesting that offering a psychological intervention for chronic pain in primary care is a fruitful avenue towards providing care to racial minorities and those with lower SES. Indeed, physicians who care for a high proportion of racial and ethnic minority patients of low SES face access issues to specialty care (Reschovsky & O’Malley, 2008). Over half (53%) of patients with chronic pain are referred for specialty pain care, although it can take several months to obtain these appointments (Schulte *et al.*, 2010). Embedding psychologists in the primary care setting has been shown to decrease waiting time and facilitate access to general behavioural health services (Pomerantz *et al.*, 2008; Miller-Matero *et al.*, 2015), and results from this study suggest that integrating a psychological intervention in primary care can also increase access for underserved patients with chronic pain.

Interestingly, results from this study showed that women were more likely to enrol in the study than men. Although this finding aligns with research showing that women are more likely than men to seek mental health services (Matheson *et al.*, 2014), other research found gender does not predict engagement in mental health treatment in a primary care environment (Miller-Matero *et al.*, 2015). Thus, perhaps there is something specific with chronic pain that could explain why women were more likely to enrol than men. Not only are women more likely to have a chronic pain condition, women with chronic pain exhibit greater levels of anxiety and depression than men (Munce & Stewart, 2007; Miller & Cano, 2009; Stubbs *et al.*, 2010). Other evidence shows women experience greater pain intensity and worse interference associated with their pain, suggesting degree of interference and discomfort may facilitate desire for treatment than men (Stubbs *et al.*, 2010; Bartley & Fillingim, 2013). Together, this evidence suggests that associated psychological sequelae and pain perception may account for the higher levels of treatment-seeking among women with chronic pain. Additionally, women are more amenable to seeking psychotherapy for mental illness than men (Holzinger *et al.*, 2012), and women have been shown to have a better response to multimodal pain treatment (e.g., being treated by physicians, psychologists, physical therapists, a nutritionist, a social worker, and relaxation therapists) as pertaining to improvements in pain ratings and pain-related disability than men (Pieh *et al.*, 2012). As such, attitudes towards psychotherapy and its perceived efficacy may account for the gender differences in enrolment observed in this study.

It is important to note that the rate of those electing to enrol in this study is lower than the randomization rate of approximately 50% found in other pilot trials (Cooper *et al.*, 2018). However, the rate that enrolled in this study was approximately three times higher than the rate that follow through with a referral to a remote behavioural health clinic (Collins & Fund, 2010), suggesting that integrating this intervention in primary care can increase engagement in a psychological intervention for chronic pain management. In addition, as mentioned, this primary care clinic is located in an urban city and serves predominantly Black patients. Although it appears that we were able to recruit participants in primary care regardless of race, we still may have had lower overall engagement due to this. Thus, methods to engage racial minorities in behavioural pain management services should continue to be explored.

There are several study limitations which should be noted. Specifically, this study was conducted as part of a pilot randomized controlled trial, and as such, the sample size is relatively small. Replication of these findings with a larger sample size is necessary. Additionally, recruitment for this study began with the primary care provider introducing it to their eligible patients. Although the primary care providers were given a brief script to use to introduce the study, we did not evaluate how the primary care providers discussed the study with their patients. Providers may have different approaches in discussing psychological treatments for pain, and some providers may have more effective approaches at engaging patients in treatment. Thus, there may have been some bias with the recruitment methods that could have led to some patients enrolling at differing rates. Lastly, we do not have record of patients’ rationales for choosing to enrol or not enrol in the study; this information would allow for an examination of both facilitators and barriers to study participation.

This study demonstrated that the primary care setting can be useful to engage patients in psychological interventions for chronic pain, especially underserved patients who may be otherwise less likely to engage in psychological services. Future research would benefit from examining whether psychological interventions to address pain-related distress and impairments in functioning are similarly effective for underserved patients. Future work could also identify whether certain approaches used by providers are more effective at engaging patients in psychological treatments for pain management. Considering this intervention was only tested in a primary care setting, it would also be beneficial to understand whether findings translate to other settings where psychologists address pain-related concerns, such as within specialty pain clinics, orthopaedics, or oncology.
